# Efficacy and safety of Shenfuqiangxin pills in complementary treatment of chronic heart failure: A protocol for systematic review and meta-analysis

**DOI:** 10.1097/MD.0000000000024531

**Published:** 2021-04-16

**Authors:** Lili Ren, Hui Guan, Guohua Dai, Wulin Gao, Jing Su

**Affiliations:** a First College of Clinical Medicine, Shandong University of Traditional Chinese Medicine; b Department of Cardiology, Affiliated Hospital of Shandong University of Traditional Chinese Medicine, Jinan, Shandong Province, China.

**Keywords:** chronic heart failure, meta-analysis, shenfuqiangxin pills, systematic review

## Abstract

**Background:**

As the last link in the chain of cardiovascular events, chronic heart failure (CHF) has high morbidity, high mortality, and poor prognosis. It is one of the main causes of death and disability worldwide. Shenfuqiangxin Pills (SFQX) is widely used as a Chinese herbal medicine (CHM) prescription for CHF, but there is still a lack of strict evidence-based medical evidence. Therefore, we make a protocol for evaluating the efficacy and safety of SFQX for CHF.

**Methods:**

According to the search strategy, randomized controlled trials (RCTs) of SFQX for CHF will be retrieved from 8 databases without limitation of publication date or language. First of all, the literature was screened according to the eligibility criteria, and use the Cochrane Collaboration's tool to assess the quality of the included literature. Then, using software for traditional meta-analysis. Finally, using GRADE method to assess the strength of recommendations.

**Results:**

This study will evaluate the efficacy and safety of SFQX for CHF, thereby providing more evidence support for clinical decision-making in CHF.

**Conclusion:**

Our research will provide more references for the clinical medication of patients with CHF.

**Protocol registration number:**

INPLASY202110019

## 1 Introduction

Heart failure (HF) is a group of clinical syndromes in which the left ventricular filling or the ability to eject blood is impaired due to abnormal heart structure or (and) function.^[1]^ The main manifestations are dyspnea, fatigue, fluid retention, among others. As the severe and terminal stage of various heart diseases, HF has a high recurrence rate and a poor prognosis, which seriously threatens people's physical and mental health.^[2]^ In developed countries, the prevalence of HF in adults is about 1% to 2%, and it gradually increases with age, and exceeds 10% in people >80 years.^[2,3]^ After being diagnosed with chronic heart failure (CHF), the rehospitalization rate was as high as 83.1%, and 42.6% were hospitalized >4 times.^[4]^ The Framingham Heart Study in the United States shows that within 5 years of the initial diagnosis of CHF, the mortality rate of patients is about 50%.^[5]^ In patients with acute myocardial infarction with CHF, the 1-year mortality rate is >50%.^[3]^ Therefore, extending the survival period of patients with CHF, improving the quality of life, improving the long-term prognosis, and reducing the mortality rate are key issues that need to be resolved.

Traditional CHF focuses on the study of hemodynamics, emphasizing the relief of symptoms in the acute phase, and the conventional treatment is mainly to cardiotonic, diuresis, and vasodilators. With the discovery of neuroendocrine mechanism, early and comprehensive intervention of cardiovascular events can improve the prognosis; conventional treatments are mostly based on neuroendocrine antagonists, such as ACEI/ARB, β-receptor blockers, and digoxin, among others. Nevertheless, the mortality and rehospitalization rate of patients with CHF remains high.

Chinese herbal medicine (CHM), as a complementary therapy, originated in ancient China and has accumulated rich experience for CHF.^[6]^ Under the guidance of the holistic concept and the thoughts of treatment based on syndrome differentiation, the treatment of CHF has the characteristics of multi-level, multi-link, and multi-target effects. A number of studies have shown that combined treatment of Chinese and Western medicine can reduce the rehospitalization rate of patients with CHF, improve the quality of life, and improve the long-term prognosis.^[7–9]^ Evidence from clinical studies of traditional Chinese medicine (TCM) is also used in the “Guidelines for the Diagnosis and Treatment of Heart Failure in China” in 2014 and 2018.^[10,11]^

Shenfuqiangxin pills (SFQX) are approved by the state, and are recommended as oral Chinese patent medicines for CHF in the 2014 and 2016 TCM diagnosis and treatment expert consensus.^[12,13]^ In some clinical studies, it has also been shown that SFQX combined with western medicine can further improve the heart function of patients with CHF, reduce myocardial damage markers and myocardial enzyme indexes, and improve the quality of life.^[14,15]^ In addition, it has also been found in some animal experiments that SFQX can reduce water and sodium retention, correct electrolyte disturbances, and inhibit autophagy and apoptosis of myocardial cells, thereby protecting the heart.^[16–18]^ It can be seen from this that SFQX may bring hope to this research field and may also arouse the interest of the international medical community. However, as there is no systematic review, it is very necessary for us to strictly review and evaluate the existing evidence so far. Therefore, the purpose of this study is to evaluate the efficacy and safety of SFQX in complementary treatment of CHF, so as to provide sufficient evidence-based medical evidence for further guidance of clinical medication and avoid unnecessary traps.

## 2 Methods

### 2.1 Research registration

Our protocol has been registered on the INPLASY. The number was INPLASY202110019 (URL= https://inplasy.com/inplasy-2021-1-0019/). We will be based on the Preferred Reporting Items for Systematic Review and Meta-Analysis Protocols (PRISMA-P), and strictly follow the requirements and conduct.^[19]^

### 2.2 Data sources and retrieval strategy

We will conduct literature search from the following databases: PubMed, EMBASE, the Cochrane Library, Web of Science, CNKI, Wan-fang Data, Chinese Biomedical Literature Database, Chinese Scientific Journal Database. There are no restrictions on publication date and language. In addition, the references listed in each included article are also manually searched.

We will retrieve the databases by combining subject words with random words. Appropriate adjustments will be made according to the grammatical rules of different databases to ensure the completeness and comprehensiveness of the search. We will first conduct a pre-search, and discuss the problems encountered in the search process with the team. After confirming that there are no problems, we will conduct a formal literature search. Taking PubMed as an example, the retrieval strategy is shown in Table 1.

**Table 1:** Retrieval strategy for PubMed.

Number	Search item
#1	Heart Failure[Mesh]
#2	Heart Failure[Title/Abstract] OR Cardiac Failure[Title/Abstract] OR Heart Decompensation[Title/Abstract] OR Decompensation, Heart[Title/Abstract] OR Heart Failure, Right-Sided[Title/Abstract] OR Heart Failure, Right Sided[Title/Abstract] OR Right-Sided Heart Failure[Title/Abstract] OR Right Sided Heart Failure[Title/Abstract] OR Myocardial Failure[Title/Abstract] OR Congestive Heart Failure[Title/Abstract] OR Heart Failure, Congestive [Title/Abstract] OR Heart Failure, Left-Sided[Title/Abstract] OR Heart Failure, Left Sided[Title/Abstract] OR Left-Sided Heart Failure[Title/Abstract] OR Left Sided Heart Failure[Title/Abstract]
#3	#1 OR #2
#4	Shenfuqiangxin Pills[Title/Abstract] OR Shenfuqiangxin[Title/Abstract] OR SFQX [Title/Abstract]
#5	randomized controlled trial [Title/Abstract] OR controlled clinical trial[Title/Abstract] OR RCT [Title/Abstract] OR randomized[Title/Abstract] OR randomly[Title/Abstract]
#6	#3 AND #4 AND #5

### 2.3 Eligibility criteria

We will formulate the eligibility criteria for this study based on the PICOS principles.

#### 2.3.1 Participants

Patients with CHF, whether diagnosed by a clinician, or by any recognized criteria diagnosis of CHF, will be included. There are no restrictions on nationality, age, sex, or race. Patients who have received acute heart failure, or patients with severe liver and kidney, or blood diseases, or malignant tumors, or other uncontrolled systemic diseases are excluded.

#### 2.3.2 Interventions and Comparators

The treatment group was given SFQX on the basis of routine western medicine of CHF. The control group was only given routine western medicine, or the same dose of placebo was given on the basis of routine western medicine. Routine western medicine mainly includes diuretics, ACEI, ARB, β-receptor blockers, ivabradine, digitalis and inotropic drugs, vasodilators, anticoagulants, etc.^[1]^

#### 2.3.3 Outcomes

The primary outcomes include TCM syndrome scores, NYHA classification; the secondary outcomes include N terminal pro B type natriuretic peptide (NT-proBNP), left ventricular ejection fraction (LVEF), quality of life, among others; the safety indicators include gastrointestinal reactions, such as nausea and vomiting, liver function indicators, allergic and other adverse reactions.

#### 2.3.4 Type of studies

Randomized controlled trials (RCTs) will be included in this study irrespective of language or publication category. Animal trials, review article and the incorrect RCT studies will be excluded.

### 2.4 Literature screening and data extraction

**Figure 1:**
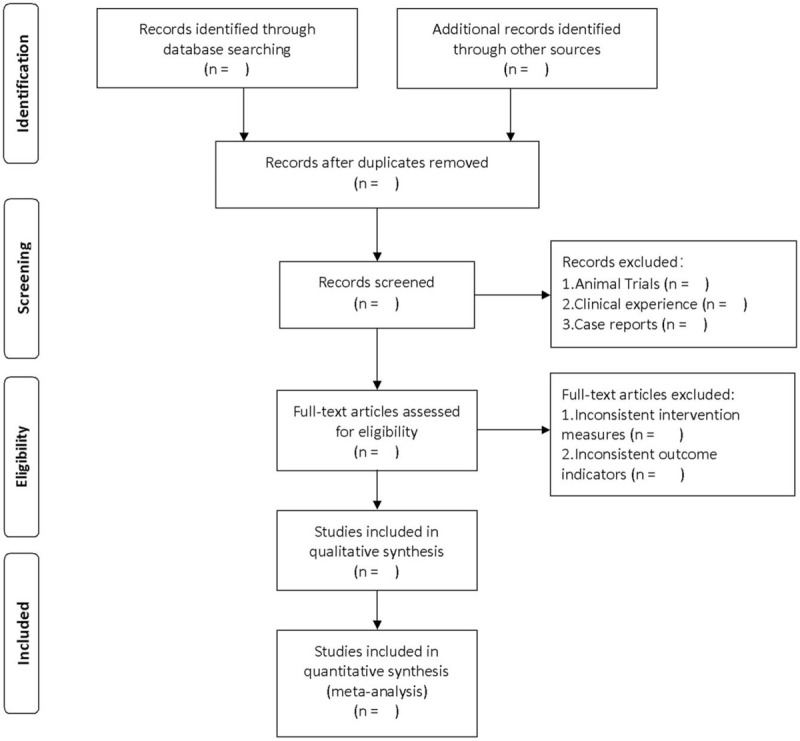
PRISMA flow chart. The figure that explains the process required to follow the PRISMA guidelines to screen the literature suitable for this study after the literature search is completed. Adapted from: Moher D, Liberati A, Tetzlaff J, Altman DG, The PRISMA Group (2009). Preferred Reporting Items for Systematic Reviews and Meta-Analyses: The PRISMA Statement. PLoS Med 6(7): e1000097. doi:10.1371/journal. pmed1000097.

Endnote X9.0 software to manage literature was used. After searching literature based on the above steps, it is imported into endnote software for literature screening. First, 2 independent researchers will conduct a preliminary literature screening based on the titles and abstracts of the included literature to eliminate duplicate and non-RCTs. Then read the full text of the remaining literature according to the previously designed principles of eligibility criteria, and finally determine the appropriate literature. When 2 researchers disagree, a third researcher will resolve it. The specific screening process is shown in Figure 1.

According to the Cochrane Handbook for Systematic Reviews of Interventions, 2 researchers independently extracted and recorded the required information from all the included literature. When 2 researchers disagree, they will discuss to reach an agreement, otherwise they will work with the third researcher to resolve. The required information mainly includes the author, publication time, study design, participants’ number, and demographic characteristics (age, sex, among others), treatment status (eg, the initial dose and treatment period of SFQX) and outcomes (eg, TCM syndrome scores, NYHA classification). If any of the above information in the included literature is incomplete, we will contact the corresponding author via email to obtain the required data.

### 2.5 Quality assessment

Two reviewers will independently assess the quality of the included literature. If there is a disagreement between 2 reviewers, the third reviewer resolves the issue. According to Cochrane Handbook V.5.2.0, Characteristics of each item will be evaluated in three categories: low, unclear, and high.^[20]^ The results of the quality assessment will be completed using software Review Manager 5.3.

### 2.6 Statistical analysis

#### 2.6.1 Traditional meta-analysis

We will execute Rev Man 5.3 and STATA 14.2 software for traditional meta-analysis. For dichotomous data, we will calculate a summary estimate with 95% confidence interval (CI) odds ratio (OR) value; for continuous data, we will calculate a summary estimate of standardized mean difference (SMD) with 95% CI, and *P* < .05 is considered statistically significant. The heterogeneity among the included literature will be assessed using the Q test method and *I*^2^ statistic method. When the Q statistic corresponds to *P* ≤ .10 or *I*^2^ >50%, it indicates that there is heterogeneity among the included literature, and assess the effect size by the random effect; on the contrary, a fixed-effect model is used.

#### 2.6.2 Subgroup analysis

Taking into account the issue of heterogeneity, we will conduct a subgroup analysis based on the specific circumstances of the included literature. If there is a problem of heterogeneity, we will conduct a subgroup analysis of age, sex, and interventions. In addition, to understand whether the LVEF will affect the efficacy of SFQX, it will be used for CHF patients with LVEF <40% and CHF with unknown LVEF the TCM syndrome scores and NYHA classification are analyzed by subgroups.

#### 2.6.3 Sensitivity analysis

This systematic review will use the method of eliminating each study one by one for sensitivity analysis. If the effective indicators (eg, TCM syndrome scores and NYHA classification) of SFQX in complementary treatment of CHF have not changed significantly, it indicates that the study is robustness. On the contrary, it is not robustness. According to the specific situation, low-quality research is excluded.

#### 2.6.4 Publication biases

Publication biases will be assessed by a funnel plot for meta-analysis and quantified by the Egger method. It should be noted that if the number of included literature is ≥10, it is appropriate to use a funnel plot. However, if the included literature is <10, it may affect the overall test power because the included number is too small, and it is difficult to accurately evaluate the symmetry of the funnel plot.

### 2.7 Evidence quality assessment

The Grading of Recommendations, Assessment, Development and Evaluation (GRADE) used to assess the quality of evidence. The quality of evidence is divided into 4 levels from 5 aspects.^[21]^

### 2.8 Ethical considerations and Dissemination plans

This study does not involve medical ethics and patients’ informed consent. And it will publish in journal papers.

## 3 Discussion

Some people say that CHF is “the last game of the heart.” From the perspective of the causes of CHF, on the one hand, as the aging of the population in various countries gradually accelerates, the incidence of various cardiovascular-related diseases is gradually increasing, such as coronary heart disease, hypertension, dyslipidemia, diabetes, obesity, and so on, and most of the end stages of these diseases end in CHF; on the other hand, with the continuous improvement of global medical technology and the change of people's medical thinking, the progress of cardiovascular disease is slow, and the survival period of patients is prolonged, which will cause the incidence of CHF to continue to increase. At present, studies have shown that the root cause of CHF is due to myocardial remodeling. Excessive activation of the sympathetic adrenergic system and the renin-angiotensin-aldosterone system leads to a variety of endogenous neuroendocrine and cytokine activation, which triggers myocardial remodeling, and long-term activation promotes myocardium remodeling, aggravating myocardial damage, and further activating neuroendocrine and cytokines, forming a vicious circle.^[22,23]^ Therefore, preventing the excessive activation of neuroendocrine and blocking myocardial remodeling is the key to treatment. However, in the current limited medical technology, the cardiac function and structure cannot be completely reversed temporarily. Some drugs and methods can only be used to prevent myocardial remodeling to a certain extent, improve or delay the deterioration of cardiac function, improve the quality of life, and improve the long-term prognosis.

SFQX are made by adding and subtracting 2 TCM preparations, the main ingredients include Ginseng (Renshen), Radix Aconiti Carmichaeli (Fuzi), White Mulberry Root-bark (Sangbaipi), Polyporus Umbellatus (Zhuling), Semen Lepidii (Tinglizi), and Rhubarb (Dahuang). In a number of clinical studies, it has been found that SFQX can inhibit the RASS system by reducing the levels of Ang II and aldosterone, thereby reversing myocardial remodeling and improving cardiac function.^[24,25]^ In addition, pharmacological studies have also shown that radix aconiti carmichaeli can warm yang and nourish fire, ginseng can nourish qi, and the combination of the 2 drugs has the effect of strengthening heart and expanding blood vessels to enhance the contractility of the heart.^[26,27]^ White mulberry root-bark and polyporus umbellatus have promoting diuresis effects and can improve heart function.^[12]^

It can be seen that it is very necessary for us to explore the complementary therapeutic effect of SFQX by formulating a strict plan, and we can make a more objective evaluation of the existing evidence and make a more accurate evaluation of the effect indicators. But at the same time, this research may also have some problems. For example, this study is an evaluation of published literature, and there may be problems such as unscientific and non-strict RCT design, resulting in uneven quality of literature research, which affects the credibility of this study, or the research results in included literature have false negatives and false positives. In any case, this study can provide reliable results for CHF, and provide strong evidence for the significant advantages of SFQX in complementary treatment of CHF.

## Author contributions

**Conceptualization:** Lili Ren, Hui Guan, Wulin Gao.

**Data curation:** Lili Ren, Hui Guan, Jing Su.

**Formal analysis:** Lili Ren, Hui Guan, Jing Su.

**Funding acquisition:** Guo-hua Dai.

**Methodology:** Lili Ren, Wulin Gao.

**Project administration:** Guo-hua Dai.

**Writing – original draft:** Lili Ren.

**Writing – review & editing:** Lili Ren, Guohua Dai.
